# SGDAN—A Spatio-Temporal Graph Dual-Attention Neural Network for Quantified Flight Delay Prediction

**DOI:** 10.3390/s20226433

**Published:** 2020-11-11

**Authors:** Ziyu Guo, Guangxu Mei, Shijun Liu, Li Pan, Lei Bian, Hongwu Tang, Diansheng Wang

**Affiliations:** 1 School of Software, Shandong University, Jinan 250101, China; guoziyu@mail.sdu.edu.cn (Z.G.); meigx@mail.sdu.edu.cn (G.M.); 2 TravelSky Mobile Technology Limited, Beijing 101318, China; bianlei@pku.edu.cn (L.B.); tanghw@travelsky.com (H.T.); dshwang@travelsky.com (D.W.)

**Keywords:** attention, air traffic data, flight delay, graph sequence, graph neural network

## Abstract

There has been a lot of research on flight delays. But it is more useful and difficult to estimate the departure delay time especially three hours before the scheduled time of departure, from which passengers can reasonably plan their travel time and the airline and airport staff can schedule flights more reasonably. In this paper, we develop a Spatio-temporal Graph Dual-Attention Neural Network (SGDAN) to learn the departure delay time for each flight with real-time conditions at three hours before the scheduled time of departure. Specifically, it first models the air traffic network as graph sequences, what is, using a heterogeneous graph to model a flight and its adjacent flights with the same departure or arrival airport in a special time interval, and using a sequence to model the flight and its previous flights that share the same aircraft. The main contributions of this paper are using heterogeneous graph-level attention to learn the influence between the flight and its adjacent flight together with sequence-level attention to learn the influence between the flight and its previous flight in the flight sequence. With aggregating features from the learned influence from both graph-level and sequence-level attention, SGDAN can generate node embedding to estimate the departure delay time. Experiments on a real-world large-scale data set show that SGDAN produces better results than state-of-the-art models in the accurate flight delay time estimation task.

## 1. Introduction

As an important issue in the air traffic system including airport management and flight scheduling, flight delay (i.e., the difference between the scheduled time and the actual time of departure or arrival) blurs the efficiency of the aeronautical system and the choice of passengers. As found in FlightStats, in September 2019, there were 489,801 flight delayed and 34,821 flights cancelled worldwide. Thus, flight delay has attracted a lot of researchers’ attention [1,2,3,4].

Actually, the reasons for flight delay are diverse. First of all, the most important and common reason is the weather. For instance, in July 2019, the flight delay rate of China was 27.58%. Among them, the flight delays due to thunderstorms accounted for 56.74% of all delays. In addition, air traffic control (ATC), mechanical failure, passengers-caused incidences, and emergencies can also cause flight delays. Meanwhile, flight delays that have occurred can be propagated through airports and airlines. In addition, the air routes between the same departure or arrival airports also affect each other within a special time interval and delay of a flight will lead to a chain reaction of these air routes.

The existing research mainly focuses on flight delay or no delay [5], the distribution of delays [1,2], and the delay propagation through airlines and airports [3,4]. But in many cases, the factors affecting flight delay are not independent. It is more useful and difficult to estimate the departure delay time with respect to real-time conditions, especially three hours before the scheduled time of departure, from which airline and airport staff can schedule flights more reasonably and passengers can reasonably plan their travel time more leisurely.

**Figure 1:**
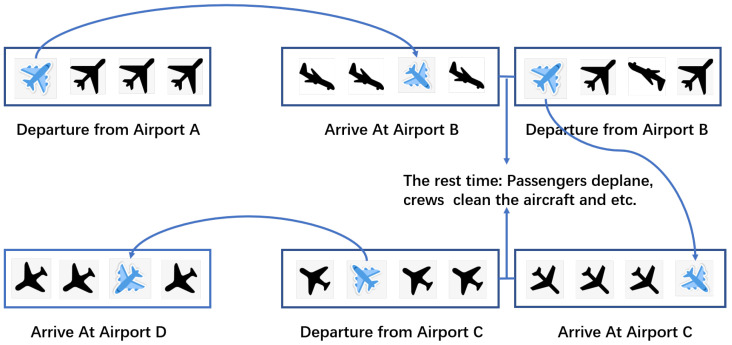
A simplified illustration of an aircraft (the blue aircraft) flying in a day, which is usually called a flight sequence.

The difficulty of this problem lies in that an air traffic network is a special and complex Spatio-temporal network. The Spatio-temporal network differs from other networks, which mainly lies in: (1) Propagation and recurrence along with links play an important role in the computations performed. (2) The representational state of the network depends not only on the node firing but also on the node’s firing time. And the air traffic network is also different from Spatio-temporal network. Firstly, as shown in Figure 1, a flight such as the blue aircraft from airport **A** to **B**, affected by not only the flights departing at airport **A**, but also the flights arriving at airport **B** in some time interval. Secondly, it has a stronger temporal dependency, and delays on a flight may be propagated throughout the day. For example, in Figure 1, due to the departure delay at airport **A**, the blue aircraft arrived in airport **B** an hour later than the scheduled time of arrival. There is no doubt that it will cause the aircraft not to be able to take off on time in airport **B**. And when the delay is too long, the airline will have to temporarily change the aircraft from airport **B** to **C** or the delay will continue to be propagated to the flight from airport **C** to **D**. Finally, Air Traffic Control (ATC) will always regulate each waypoint in real-time. The above makes the flight departure a very complex decision-making process.

In addition, many emergencies cannot be obtained in time, such as temporary air traffic control and mechanical failures. Some necessary information is not accurate enough at three hours before the scheduled time of departure, such as weather forecasting. Therefore, it is necessary but difficult to predict the flight departure delay time from these limitations.

**Figure 2:**
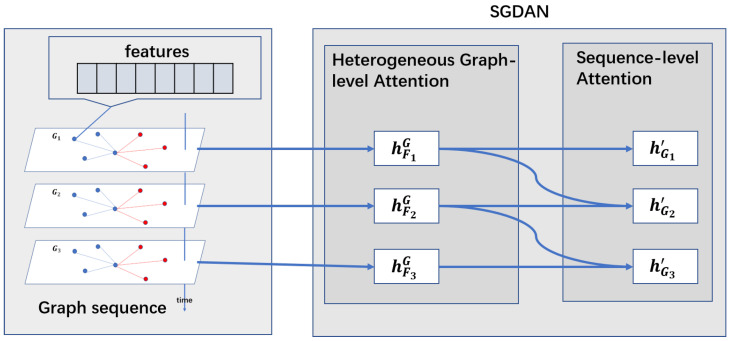
The Spatio-temporal graph dual-attention neural network. $h_{F_{n}^{G}},n\in \left\{1,2,3\right\}$ are the embedding of flight $F_{n}$ in the whole heterogeneous graph. And $h_{G_{n}^{\prime }},n\in \left\{1,2,3\right\}$ are the final embedding of flight $F_{n}$ which are aggregated by all heterogeneous graph and the graph sequences.

In this paper, we propose a novel model, named Spatio-temporal Graph Dual-Attention Neural Network (SGDAN), to model the air spatio-temporal network. And we use SGDAN to predict the flight departure delay time at the three hours before the scheduled time of departure. The whole end-to-end framework is shown in Figure 2.

We first model the complex air spatio-temporal network as a graph sequence. As shown in Figure 2, in an air traffic network, a graph sequence is a sequence of heterogeneous graphs, which models all flights made by an aircraft in a day in chronological order. Among them, a heterogeneous graph is a network constructed by the current flight and its neighbor flights. In each heterogeneous graph, a node denotes a flight and an edge between two flights denotes that the two flights have a certain relation based on a meta-path. There are two meta-paths, that is, FAF (flight-the same arrival airport-flight) or FDF (flight-the same departure airport-flight) in our dataset. Then we take weather conditions, aircraft type, aircraft age and other factors as the inherent attributes of the nodes in the graph.

Then the SGDAN is developed to embed the graph sequence we defined. And we can use the embedding to predict the departure delay time. First, we use heterogeneous graph-level attention to embed the heterogeneous graph and we use a soft-gate to control multi-head. Specifically, we use multi-head attention to learn the importance of each neighbor flight based on different meta-paths (FAF or FDF) and use the importance to aggregate the features of a node (flight) and the neighbor flights. And different heads extract features from different views to get different weights, so we use a small convolutional sub-network to compute a soft gate at each attention head to control its importance. Then, we get two new features through the two meta-paths. We also use attention to aggregate the two new features obtained through different meta-paths. Finally, we use sequence-level attention to learn the importance of previous flights and aggregate the features between a node (flight) and its previous nodes (flights) that share the same aircraft. Based on the heterogeneous graph-level and sequence-level attention, SGDAN can get the optimal combination of the heterogeneous graph and the flight sequence, which enables the learned node embedding to better capture the complex Spatio-temporal network information in the graph sequence. We will describe it in Section 3 in detail.

We validate SGDAN by predicting the departure delay time on a real dataset provided by Umetrip which brings together information on all flights from all airports and airlines. The results show that SGDAN produces an effective result in predicting the departure delay time. The results are better than state-of-the-art models in the accurate flight delay time estimation task.

In summary, this paper makes the following contributions:

1. It abstracts the complex air spatio-temporal network into graph sequences, which uses graphs to model spatial dependencies, and sequences to model temporal dependencies.
2. Based on the abstraction, it proposes a novel model, SGDAN, which embeds the graphs by using heterogeneous graph attention and a soft gate to control multi-head. Through the heterogeneous graph-level attention, SGDAN embeds the impact of other flights with the same departure or arrival airports effectively. Then SGDAN uses sequences-level attention to embed the flight sequences which integrates the impact of the previous flights that share the same aircraft.
3. In predicting the flight departure delay time task, SGDAN gets a better result compared with state-of-the-art models. It proves that it is feasible and effective to abstract the spatio-temporal network into graph sequences and then construct a graph neural network in spatio-temporal networks.

## 2. Related Work

### 2.1. Flight Delay Prediction

Researchers have studied flight delays based on various views, such as aircraft taxiing delay [6,7], distributions of departure or arrival delays [1,2], and how delay propagated through airports and airlines [3,4].

Specifically, as for aircraft taxi-out, Poornima Balakrishna et al. used reinforcement learning algorithms for predicting the aircraft taxi-out time [6]. The influencing factors included passenger demand, fares, flight frequency, aircraft size and so forth. Ludovica Adacher et.al. dealt with the problem of routing and scheduling aircraft ground movement operations to minimize the total routing taxiing delay [7].

As for distributions of departure, en route and arrival delays, Eric Mueller et al. used probabilistic models to analyze aircraft arrival and departure delay characteristics [1] and Yufeng Tu et al. used a statistical approach to identify and study major factors that influence flight departure delays, and developed a strategic departure delay prediction model which employed non-parametric methods for daily and seasonal trends [2].

As for airport capacity, Loan Le et al. studied optimum airport capacity utilization under congestion management to reduce flight delays [8]. Mayara Condé Rocha Murça et al. presented a data-driven framework to identify, characterize, and predict traffic flow patterns in the terminal area of multi-airport systems toward improved capacity planning decision support in complex airspace [9].

In addition, other researchers have analyzed flight delays for different scopes. Juan Jose Rebollo et al. focused on airports to predict departure delays for all flights, while considering delays in all airlines and air lines indifferently [10]. Other studies have investigated the efficiency of all airlines based on delays [11,12].

In recent years, some researchers studied how delay propagated through airports and airlines of flight delays. Du Wen-Bo et al. built a delay causality network (DCN) based on the Granger causality test to understand the mechanism of flight delay propagation at the system-level [11,12]. Weiwei Wu et al. captured the interdependency among the sequences of flight delays due to airline operations in airports, weather, and air traffic control conditions [4]. Weiwei Wu et al. used a set of real airline data and developed an enhanced Delay Propagation Tree model with Bayesian Network (DPT-BN) whose results showed that flights have non-homogeneous delay propagation effects [13].

In addition, a few models are able to predict accurate flight delay time [14]. But the overfitting of their results is severe and the performance is poor. G. Gui et al. built a dataset from automatic dependent surveillance-broadcast (ADS-B) messages and other information such as weather conditions, flight schedule, and airport information. Then, it used machine learning to predict accurate delay time [15]. Then, the same authors also proposed a gradient boosting decision tree (GBDT) based on the same dataset for generalized flight delay prediction [16].

### 2.2. Graph Neural Networks

As early as in 2009, Scarselli Franco et al. proposed the Graph Neural Networks (GNN) model [17] which first extended neural networks to the graph domain. The inputs of GNN include node features and the relations between the nodes. Its target is to learn an embedding that contains nodes’ features and the information of nodes’ neighbors.

In the following years, there was a lot of research on improving GNN. Among them, some researchers use convolutional networks to improve GNN. On the one hand, Joan Bruna et al. define the convolution operation in the Fourier domain that computes the eigendecomposition of graph’s Laplacian and proposed spectral networks [18]. And some research constantly optimizes and accelerates the calculation [19,20,21]. In the end, Graph Convolutional Networks (GCN) [22] becomes the most popular algorithm which is used for semi-supervised classification and FastGCN [23] optimizes GCN [22] for large-scale graphs. On the other hand, some research defines convolutions directly on the graph with operations on spatially-close neighbors and constantly optimize it [24,25,26,27]. Finally, William L Hamilton et al. proposed GraphSAGE [28], a general inductive framework that generated embedding by sampling and aggregating features from a node’s local neighbors. Rex Ying et al. extended this model for web-scale recommender systems [29].

Some researchers use attention mechanisms to improve GNN. Ashish Vaswani et al. show that self-attention can improve RNN-based or convolution-based models and build a powerful model that obtains access to the most advanced performance in machine translation tasks [30]. Inspired by this, GATs [31] was proposed and address several key challenges of spectral-based graph neural networks simultaneously. On this basis, Jiani Zhang et al. proposed GaAN [32], which adopted a multi-head attention-based aggregator with additional gates on the attention heads. In addition, other researchers improve GNN [33] based on GAN [34].

However, these simple graph-based GNN models cannot effectively model the flight delay system which includes different features, co-competitive graphs with the same departure or arrival airport and the flight sequence as described in Section 1.

In addition, some researchers have begun to use GNN’s ideas to solve more complex graph problems. HAN [35] uses the attention mechanism to embed the heterogeneous graphs. HAN includes two attention levels: it first learns the importance between a node and its meta-path based neighbors and on this basis learns the importance of different meta-paths. GMNN [36] combines the advantages of graph neural networks and statistical relational learning models. In the E-step, one graph neural network learns effective object representations via nodes’ features and their neighbors. In the M-step, another graph neural network is used to model the local label dependency.

Recently, lots of researchers have studies dual-attention graph neural networks [37,38] and developed a serious of application for general spatio-temporal network in different urban traffic scene [39,40].

Inspired by HAN [35] and GMNN [36], we try to solve this complicated graph problem through multiple levels (steps). Also, inspired by GaAN [32], we use a soft gate at each attention head to control its importance. The gate-generation network acts as a high-level controller that determines how to aggregate the features extracted by the attention heads. Thus, we abstract the complex spatio-temporal network into graph sequences. And based on the graph sequences, we proposed SGDAN.

## 3. Model: SGDAN

Due to the complexity of the air spatio-temporal network, we first model the complex air traffic network as graph sequences, thereby we can model the special network to predict the flight departure delay time at three hours before the scheduled time of departure. The notations are shown in Table 1.

**Figure 3:**
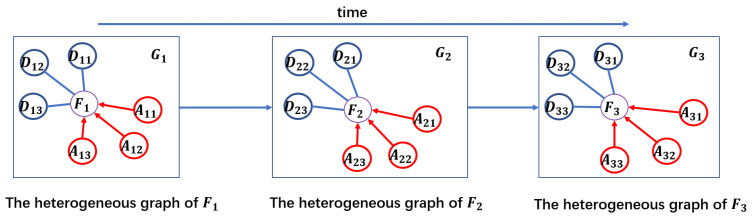
A graph sequence based on the scenario shown in Figure 1. $F_{n},n\in \left\{1,2,3\right\}$ denotes the *n*-th flight made by the blue aircraft. For example, $F_{1}$ denotes the flight from airport **A** to airport **B** made the blue aircraft. $D_{nm},n,m\in \left\{1,2,3\right\}$ are the flights with the same departure airport as $F_{n},n\in \left\{1,2,3\right\}$ and $A_{nm},n,m\in \left\{1,2,3\right\}$ are the flights with the same arrival airport as $F_{n},n\in \left\{1,2,3\right\}$. For example, $D_{1}1$ denotes the flights departing from airport **A** except for the blue filights. And $A_{1}1$ denotes the flights arriving at airport **B** except for the blue filights.

**Graph sequence**. As shown in Figure 3, in an air Spatio-temporal network, a graph sequence, denotes as $S=\{G_{1},G_{2},\cdots ,G_{N}\}$, is a sequence of *N* graphs, means that an aircraft makes *N* flights in a day and $G_{n},n\in \left\{1,2,\cdots ,N\right\}$ is a heterogeneous graph of the current flight $F_{n}$ in the networks where a node represents a flight and an edge represents the relationship, that is, FAF (flight-the same arrival airport-flight) or FDF (flight-the same departure airport-flight), between two flights.

Firstly, as shown in Figure 1, a flight such as the blue aircraft from airport **A** to **B**, affected by not only the flights departing at airport **A**, but also the flights arriving at airport **B** in a special time interval. We firstly use a heterogeneous graph to model spatial dependencies. Specifically, we use a node to represent a flight and use an edge to represent the relationship between two flights. In the scenario shown in Figure 1, the relationships between two flights include FAF (flight-the same arrival airport-flight) and FDF (flight-the same departure airport-flight). Thus, we can construct a heterogeneous graph, denotes as G=(V,E,M,L), includes a node set *V*, an edge set *E*, a meta-path set *M* and a nodes’ label set *L*. Here a node denotes a flight and an edge between two flights denotes that the two flights have a certain relation based on a meta-path such as the same arrival airport. For example, in Figure 1, the relation can be the same arrival airport and the same departure airport. The edge set through a map function Φ:E→M, *M* is the meta-path set. In this paper, two flights can be connected via two meta-paths, that is, FAF (flight-the same arrival airport-flight) or FDF (flight-the same departure airport-flight). In addition, in Figure 3, according to the scenario in Figure 1, we only draw three neighbors for each flight based on each metapath, that is, m∈{1,2,3}. However, in our work, we build a graph sequence for each aircraft by day. And for each flight, according to the existing research [6,7] and the field experience of the data providing company, we used the time window, within half an hour before and after departure or arrive, to obtain neighbors with the same departure airport or the same arrival airport.

Secondly, as shown in Figure 1, an aircraft that is, the blue aircraft can make many flights in a day. Meanwhile, the previous flight’s delay will cause a chain reaction as mentioned above. For example, in the scenario shown in Figure 1, if the blue aircraft arrived at **B** airport two hours late due to the weather condition, it would not be able to take off at **B** airport on time, which would cause subsequent flights to be delayed. After modeling the spatial dependencies of a flight with a heterogeneous graph, we use sequences to model this temporal dependence. That is, we use a sequence of heterogeneous graphs to model all flights made by an aircraft in a day in chronological order. In general, we use $\left\{G_{1},G_{2},\cdots ,G_{N}\right\}$ represents the graph sequence where $G_{n},n\in \left\{1,2,\cdots ,N\right\}$ represents the heterogeneous graph of the n-th flight $F_{n}$ that the aircraft made.

**Figure 4:**
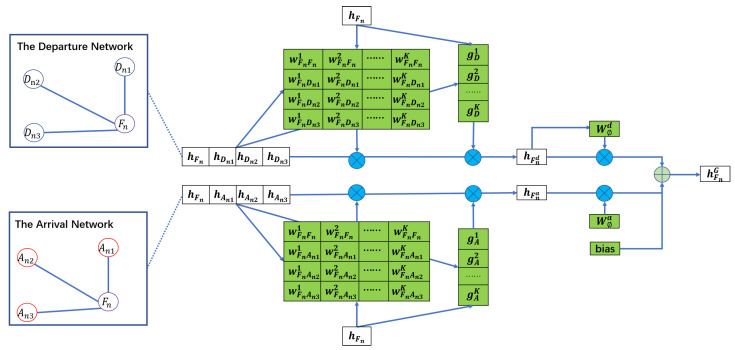
The heterogeneous graph-level attention in Spatio-temporal Graph Dual-Attention Neural Network (SGDAN) where the green background vectors need to be learned with the Equation (1). The departure network and the arrival network are constructed by different meta-paths, i.e., FDF and FAF respectively.

**Figure 5:**
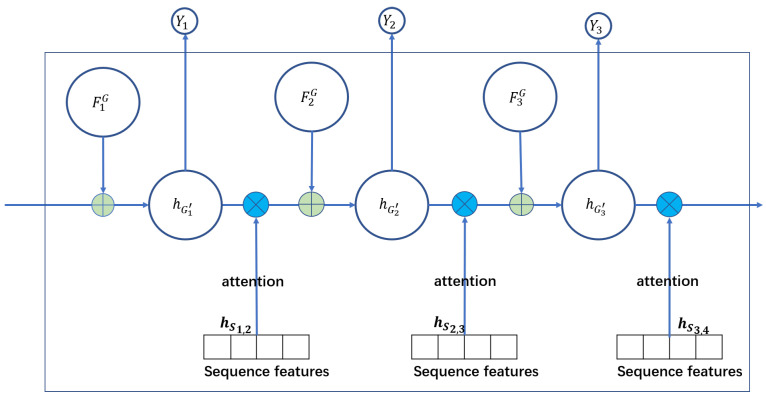
The sequence-level attention in SGDAN. And the details of $F_{n}^{G},n\in \left\{1,2,3\right\}$ are the embedding by the heterogeneous graph-level attention shown in Figure 4. $h_{G_{n}^{\prime }},n\in \left\{1,2,3\right\}$ are the final embedding of flight $F_{n}$ which are aggregated by all heterogeneous graph and the graph sequences. And $Y_{n},n\in \left\{1,2,3\right\}$ are the prediction results obtained by embedding.

Based on graph sequence, we propose a spatio-temporal graph attention neural network, named SGDAN. SGDAN follows a hierarchical structure: heterogeneous graph-level attention → sequence-level attention neural networks. In Section 1, we have presented the whole end-to-end framework in Figure 2. And we will describe the details of the heterogeneous graph-level attention in Figure 4 and the details of the sequence-level attention in Figure 5. Firstly, we discuss the hierarchical attention structure.

### 3.1. Heterogeneous Graph-Level Attention

SGDAN first learns the impact of space factors which play a different role and show different importance on flights. In this paper, based on different meta-paths, that is, FDF and FAF described in Section 1, a flight has different neighbors and can be constructed as different networks. As shown in Figure 4, $F_{n}$ denotes a flight, $\left\{D_{n1},D_{n2},D_{n3}\right\}$ are its neighbors with the same departure airport and $A_{n1},A_{n2},A_{n3}$ are its neighbors with the same arrival airport, which compose the departure network and the arrival network respectively.

#### 3.1.1. Nodes Aggregating Based on Different Meta-Path

First, as shown in Figure 4, in the departure network, we use the multi-head attention [31] to learn the weight between $F_{n}$ and its neighbors, that is, $F_{n}$, $D_{n1}$, $D_{n2}$, $D_{n3}$. In other words, the attention can learn the importance which means how important the neighbors, that is, $F_{n}$, $D_{n1}$, $D_{n2}$, $D_{n3}$, will be for node $F_{n}$. For convenience, we use $i,i\in \{F_{n},D_{n1},D_{n2},D_{n3}\}$ to represent the neighbors of $F_{n}$ in the following. Thus the multi-head attention can be formulated as follows:

(1) $$W_{F_{n}i}^{k}=att_{head}\left(h_{F_{n}},h_{i},k\right),i\in \left\{F_{n},D_{n1},D_{n2},D_{n3}\right\},k\in \left\{1,2,\cdots ,K\right\},$$

where $W_{F_{n}i}^{k}$ means the importance that node $i,i\in \{F_{n},D_{n1},D_{n2},D_{n3}\}$ will be for node $F_{n}$ by *k*-th head.

Different heads extract features from different views to get different weights. Together with that in the air Spatio-temporal network, different features such as visibility and temperature have a different impact on flights. So, inspired by Reference [32], SGDAN uses a small convolutional sub-network to compute a soft gate at each attention head to control its importance. We then can aggregate the features of $F_{n}$ and $\left\{D_{n1},D_{n2},D_{n3}\right\}$ via the weights obtained by the attention, the formula is:

(2) $$h_{F_{n}^{d}}=\sigma \left(\overset{K}{\underset{k=1}{\left|\right|}}g_{D}^{k}\sum_{i}W_{F_{n}i}^{k}h_{i}\right),i\in \left\{F_{n},D_{n1},D_{n2},D_{n3}\right\},$$

where $g_{D}^{k}=\psi \left(F_{n},i\right)$, ψ is a convolutional network that takes the $F_{n}$ and neighboring node features as the input to generate the gate values, σ is softmax activation function, || represents concatenation, $h_{i}$ is the features of *i* and $g_{D}^{k}$ is a scalar, the gate value of the *k*-th head.

Like the departure network, in the arrival network, we repeat the learning to aggregate the features of $F_{n}$ and $\left\{A_{n1},A_{n2},A_{n3}\right\}$, thereby we can get a new aggregation feature of $F_{n}$, that is, $h_{F_{n}^{a}}$.

#### 3.1.2. Different Meta-Path Aggregating

Generally, a node (flight) in the departure network and the arrival network, contains different information, thus $h_{F_{n}^{d}}$ and $h_{F_{n}^{a}}$ can only reflect the node’s information from one aspect. So, we use another attention $q_{1}$ to learn the importance of each meta-path, what is, measure the importance of $F_{n}^{d}$ and $F_{n}^{a}$. The importance of each meta-path, denoted as $W_{\Phi }$, is shown as follows:

(3) $$W_{\Phi }^{d}=q_{1}^{T}\cdot h_{F_{n}^{d}},$$

(4) $$W_{\Phi }^{a}=q_{1}^{T}\cdot h_{F_{n}^{a}},$$

which can be interpreted as the contribution of the meta-path Φ for a specific task. With the learning weights as coefficients, we can fuse $h_{F_{n}^{d}}$ and $h_{F_{n}^{a}}$ to obtain the heterogeneous graph embedding $h_{F_{n}^{G}}$ as follows:

(5) $$h_{F_{n}^{G}}=\sigma \left(W_{\Phi }^{d}h_{F_{n}^{d}}+W_{\Phi }^{a}h_{F_{n}^{a}}+b\right),$$

where σ is softmax activation function and **b** is the bias vector.

### 3.2. Sequence-Level Attention

After the heterogeneous graph-level attention, SGDAN has embedded the flight with spatial factors. In other words, the embedding can be used to predict flight delays if we do not consider the impact of temporal factors that is, the previous flights. However, in fact, the delay of a flight often has a significant impact on its subsequent flights, especially when the delay is relatively long or the rest time between the flight $F_{n-1}$ and its subsequent flight $F_{n}$ is relatively short.

Therefore, in order to measure the impact of the previous flights that share the same aircraft in different conditions, we design the sequence-level attention. To be specific, as shown in Figure 5, for every flight (node) $F_{n}$, we use the sequence-level attention to learn the impact of its previous flight $F_{n-1}$. Here we use sequence features to learn. The sequence features, denote as $h_{s_{n-1,n}},n\in \left\{1,2,\cdots \right\}$ contain the features, such as the distance between the arrival airport of $F_{n-1}$ and the arrival airport $F_{n}$, the rest time between the flight $F_{n-1}$ and its subsequent flight $F_{n}$. These features are closely related to the impact of the previous flights, but not reflected in $h_{G_{n}}$. The impact of the previous flight $F_{i}$, denotes as $W_{G_{n-1,n}}$, is shown as:

(6) $$W_{G_{n-1,n}}=q_{2}^{T}\cdot h_{s_{n-1,n}},$$

where $q_{2}$ is an attention vector. With the learning impact coefficients, we can fuse the flight embedding $h_{F_{n}^{G}}$ and its previous flight embedding $h_{F_{n-1}^{G}}$ to obtain the final embedding $h_{G_{n}^{\prime }}$:

(7) $$h_{G_{n}^{\prime }}=\sigma \left(W_{G_{n-1,n}}h_{F_{n-1}^{G}}+h_{F_{n}^{G}}+b\right),$$

where σ is softmax activation function and **b** is a bias vector. It should be pointed out that when n=1, $h_{F_{G}^{0}}$ is a zero vector because there are no previous flights for the first flight every day that shares the same aircraft.

The final embedding $h_{G_{n}^{\prime }}$ is aggregated by all heterogeneous graph embedding. In this way, the final embedding $h_{G_{n}^{\prime }}$ not only includes the impact of the current conditions on the flight but also includes the impact of its previous flights on it. Then we can apply the final embedding to specific tasks and design different loss functions which we will describe in the next section.

## 4. Experiments

To evaluate the performance of SGDAN in estimating the departure delay time, we take several semi-supervised classification experiments on our data set. Furthermore, a set of comparative experiments are conducted with the same experimental conditions to show the superior performance of our proposed model. We implement SGDAN with Tensorflow 1.9 (https://www.tensorflow.org) and Python 3.6 (https://www.python.org), and train the model with an NVIDIA GeForce RTX 2080 Ti GPU. The experiments are completed on Windows 10 with 24 Intel Xeon Gold CPUs.

### 4.1. Data Set

The dataset we used is provided by Umetrip that brings together information on all flights from all airports and airlines. The dataset we used in this paper includes 2834 aircraft flying for a week, involving 77,924 flights. The data on every flight includes the departure airport, the arrival airport, the scheduled time of departure, the scheduled time of arrival, the actual time of departure, the actual time of arrival, the aircraft type and age, and weather data (involves temperature, visibility, wind speed, cloud and weather phenomenon) for relevant dates at the airports.

#### 4.1.1. Data Preprocessing

There are some dirty data in the original data set. For example, some required data such as the scheduled time is null, and some data is wrong. We manually proofread these data, correct the data that can be corrected, and discard the data that cannot be corrected. In addition, there are some flights that will stopover somewhere. These flights are still affected by airports, airlines, and passengers during the stopover and still affect other flights. Therefore, we divide these stopover flights into multiple flights for processing. That is, every time a flight stops, we treat it as a new one.

After processing, our valid dataset includes 74,297 flights. Here we use 40,000 flights for training, 2000 for validating and the rest for testing.

We compute the departure delay time by calculating the difference between the actual time of departure and the scheduled time of departure. Then we transform these time slots into bins of constant values (30 min). To fit the learning process of SGDAN, these constant values are considered as the predicted representative labels in this experiment.

#### 4.1.2. Features.

Based on the existing paper [5] and the experience from the data-providing company, that is, Umetrip, we extracted some detailed features. Before giving the definition of the final features, we first give some definitions of different attributes.

1. **weather.**
  (8) $$w=[w_{t},w_{v},w_{s},w_{c},w_{p}],$$
  where $w_{t},w_{v},w_{s},w_{c}$ and $w_{p}$ are temperature, visibility, wind speed, cloud, and weather phenomenon.
2. **aircraft.**
  (9) $$a=[a_{t},a_{g}],$$
  where $a_{t}$ and $a_{g}$ are the aircraft type and age.
3. **time.**
  (10) $$t=[t_{w},t_{d},t_{m},t_{s}],$$
  where $t_{w},t_{d},t_{m}$ and $t_{s}$ are the day of week, day of month, month and the season.
4. **air routes.**
  (11) $$r=[r_{d},r_{a},r_{b},r_{de},r_{da},r_{ar}],$$
  where $r_{d},r_{a},r_{b},r_{de},r_{da}$ and $r_{ar}$ are the crow-fly distance, the azimuth angle of the routes, backlog rate at the departure airport, departure rate at the departure airport, delay rate at the arrival airport and acceptance rate at the arrival airport. Among them, $r_{d}$ and $r_{a}$ are calculated from latitude and longitude of the departure airport and arrival airport, the others are calculated by the following formulas:
  (12) $$r_{b}=\frac{N(DN)}{N(DA)},$$
  where N(DN) is the number of flights that should have departed but did not actually depart in the time window, and N(DA) is the number of flights that should have departed in the time window.
  (13) $$r_{de}=\frac{N(FD)}{N(DV)},$$
  where N(FD) is the number of flights that have departed in the time window, and N(DV) is the mean of historical departures in the time window.
  (14) $$r_{da}=\frac{N(AN)}{N(AA)},$$
  where N(AN) is the number of flights that should have arrived but did not actually arrive in the time window, and N(AA) is the number of flights that should have arrived in the time window.
  (15) $$r_{ar}=\frac{N(FA)}{N(FV)},$$
  where N(FA) is the number of flights that have arrived in the time window, and N(FV) is the mean of historical arrivals in the time window.

From the above, we can define our features of every fight, that is, $h_{i},i\in \left\{F_{n},D_{n1},D_{n2},D_{n3}\right\}$ in Equation (2), as

(16) $$h_{i}=\left[w_{d},w_{a},a,t,r\right]$$

where $w_{d}$ is the weather w of the flight *i*’s departure airport and $w_{a}$ is the weather w of the flight *i*’s arrive airport. **a**, **t** and **r** are the aircraft vector, time vector, air routes vector we have defined before.

The sequences features, that is, $h_{s_{n-1,n}},n\in \left\{2,3,\cdots \right\}$ are as follows:

1. **Crow-fly distance.** A crow-fly distance of flight $F_{n-1}$, calculated from latitude and longitude of its departure airport and arrival airport.
2. the rest time between the previous flight $F_{n-1}$ and the flight $F_{n}$.
3. the distance between the arrival airport of the previous flight $F_{n-1}$ and the departure airport of the flight $F_{n}$.

Among these features, we use one-hot for discrete features such as weather phenomena and normalize continuous features such as crow-fly distance. Then we construct the SGDAN based on the flights with the same departure airport and the flights with the same departure airport in a special time interval (such as one hour), as well as the flights that share an aircraft on the same days.

### 4.2. Experimental Evaluation

We define the problem of estimating the departure delay time as a piecewise classification [4] rather than regression. There are two reasons: Firstly, delays that cannot be predicted from the data set, such as delays caused by human factors or mechanical failures, will cause excessive differences in regression. Secondly, passengers and flight dispatchers usually do not need the exact delay time. In fact, a smaller delay interval can meet the demand. In our dataset, we classify each flight’s departure delay time into 30 min.

**Figure 6:**
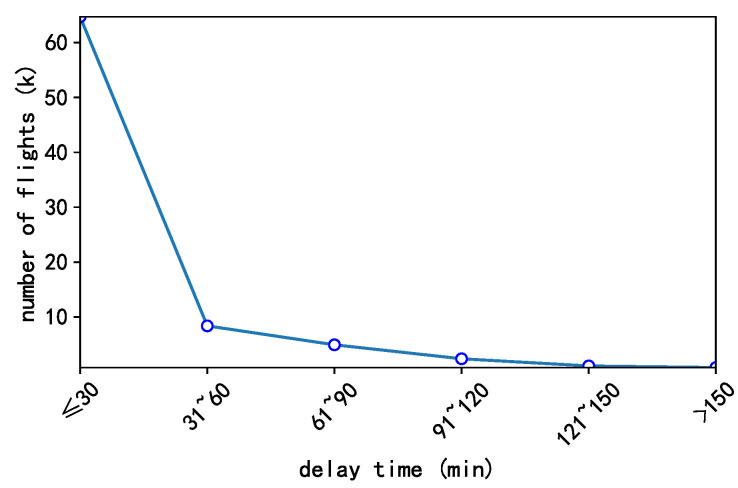
The departure delay time distribution in our dataset.

However, as mentioned in the existing paper [2], the departure delay distribution is fat-tailed distribution. And our dataset does obey the fat-tailed distribution as shown in Figure 6. In other words, the classes are imbalanced and it is a universal problem in flight delays. So we use a weighted cross-entropy as the loss function:

(17) $$Loss=-\sum_{i=1}^{N}w_{i}L_{i}\ln\left(Y_{i}\right),$$

where $w_{i}$ is the weight of the delay time class, $L_{i}$ and $Y_{i}$ are the labels and embedding of flight *i*.

Due to the fat-tail distribution of the departure delay time as Figure 6, in our experiments, we use Precision, Recall and $F_{1}$ to evaluate the performance of the baseline model and SGDAN. In addition, whether the boundary value of each class, such as a delay of 29 min, is predicted to be in the critical class (delay 30–60 mins) is also correct. The range of this boundary value is five minutes before and after the threshold, that is, 25–35, 55–65, 85–95 and 115–125 min.

As for the weight of the delay time, $w_{i}$, there are usually two methods to calculate it:

(18) $$w_{i}=\left(P+N\right)/P$$

(19) $$w_{i}=N/\left(P+N\right),$$

where *P* and *N* are the number of positive cases and negative cases of the class *i* in our dataset.

We conducted experiments with Equations (18) and (19) respectively, and the results are shown in Table 2 and Table 3.

From Table 2 and Table 3, we can find that Table 2 shows a better precision performance of SGDAN and Table 3 shows a recall performance of SGDAN. This is because Equation (19) gives a higher weight to fewer categories in the weighted cross-entropy. However, for passengers, airlines and airports, it is better not to give early warnings of delays than to give false early warnings. Therefore, getting better precision is always more important than recall. Thus, in our paper, we use Equation (18) to calculate the weighted cross-entropy, that is, Equation (17).

### 4.3. Baselines

We compare SGDAN with two state-of-the-art models [15,16]. In References [15,16], the authors also define the predicted flight delay time as a classification problem: include binary categories, three categories, and four categories. Because these two papers [15,16] have no released data set, and due to the particularity of the data set, the method cannot be reproduced. In this situation, we only doing the same tasks to compare SGDAN with these methods. Meanwhile, we use the same evaluation as the two methods, that is, accuracy and precision [15,16] in the comparison.

#### 4.3.1. Binary Categories

Binary-Categories is to predict flight delay or no delay. In addition to the methods [15,16], we also compared other methods for the same task. Zhongbin Li et al. and Young Jin Kim et al. use long short term memory (LSTM) network to predict flight delay [41,42] and Nuno Fernandes et al. extracts a series of features and uses machine learning, that is, Neural Network (NN), Support Vector Machines (SVM) and Random Forest (RF) to predict [5]. These models were evaluated with accuracy in the paper. The results are shown in Table 4.

From Table 4, we can see that SGDAN achieves the best performance. Comparing with Reference [5], SGDAN’s results far exceed it. It is due to the fact that SGDAN not only takes into account the delayed propagation in the flight sequences, but also captures the spatial factors which impact flights. Meanwhile, Zhongbin Li et al. [41] and Young Jin Kim et al. [42] also take into account the delayed propagation in the flight sequences but it does not model the spatial factors, so the results are still worse than SGDAN. The results demonstrate that the graph sequence we proposed and the model, SGDAN, proposed for this are effective.

#### 4.3.2. Three Categories

Three-Categories divide the flight delay time into no delay, delay within 2 h and delay over 2 h. The results are shown in Table 5. We first give the precision as the methods [15,16] and finally give a total accuracy.

#### 4.3.3. Four Categories

Four-Categories is to divide the flight delay time into no delay, delay within 1 h, delay within 2 h and delay over 2 h. We also first give the precision as the methods [15,16] and finally give a total accuracy.

From Table 5 and Table 6, we can find that SGDAN performs better than others, especially when predicting the long delays, that is, delay time over 2 h. Meanwhile, SGDAN can achieve better accuracy with better precision, which means that our recall value is much higher than other methods. As we mentioned above, it is worse to give false early warnings of delays than not to give early warnings. Thus, SGDAN is more efficient and practical. In addition, by comparing Table 5 and Table 6, we find that the performance of these two methods [15,16] in the Four-Categories is much worse than the Three-Categories, while the performance of SGDAN almost remain the same level. Obviously, our approach makes it easier to distinguish between different delay periods. This is due to the more detailed feature, and the special loss function in our model. Like in the methods [15,16], compared with the weighted cross-entropy loss function, the data noise is increased by using a random under-sampling strategy to obtain fairer prediction results. Besides, both the spatio-temporal factors and the propagation of delays in space-times are considered, which allows SGDAN to better learn how long each flight would be delayed and make SGDAN have a stronger modeling ability for delays in different time periods.

### 4.4. Discussion

Combined with the analysis of Table 2, we find a fact that the flights with the delay time less than 30 min account for 80%, so it is easy for the model to fully learn this category and get better results. Besides, delays of unpredicted flights are mostly caused by factors not clearly declared in the dataset such as air traffic control and mechanical failure. And the delay time is usually very long in these two categories. Because of once such delay occurs, it is often difficult to be compensated through the subsequent flights, which cause a long delay for all subsequent flights sharing the aircraft. We find such reasons cause a large proportion of long-delayed flights. Due to the fact that missing air traffic control data in our dataset, the features of this category are difficult to learn. However, as we mentioned above, comparing with the existing methods, even in the absence of these data, our model still got the best results so far.

The experiments prove that it is feasible to abstract the spatio-temporal network into graph sequences and then construct graph neural networks to learn.

## 5. Conclusions

This paper first models the complex air spatio-temporal network as graph sequences which uses graphs to model the spatial dependence and uses sequences to model the temporal dependence. Then, this paper proposes a novel model, SGDAN, which embeds the graph sequences. SGDAN uses graph-level attention to embed the heterogeneous graph firstly. Then, SGDAN uses sequence-level attention to aggregate the features between a node (flight) and the previous nodes (flights) that share the same aircraft. We conducted extensive and comprehensive experiments on a real dataset to evaluate the performance of SGDAN. The experimental results demonstrate that SGDAN significantly outperforms state-of-the-art models in two alternative tasks.

SGDAN is not limited to the prediction of flight delays. The idea of modeling the spatio-temporal network as a graph sequence and then using a graph neural network to learn can be widely extended to other similar spatio-temporal networks or used to study other flight problems. It will be our next work.

There are several potential improvements and extensions that can be addressed as future work. SGDAN has the general limitation of Graph Neural Network, what is, stacking multiple layers will result in over-smoothing and all vertices will converge to the same value. Therefore, training with fewer neural layers will lead to poor results when an aircraft makes too many flights. But in fact, an aircraft rarely makes more than 4 flights. In other words, fewer passengers will experience poorer services when others will have a better experience.

## Figures and Tables

**Table 1:** List of notational conventions.

Variables	Description
*V*	A node set
*E*	An edge set
*M*	A meta-path set, include FAF and FDF
*L*	The nodes’ label set
$G_{n}=\left\{V,E,M,L\right\}$	A heterogeneous graph, n∈{1,2,⋯,N}
$S=\{G_{1},G_{2},\cdots ,G_{N}\}$	A graph sequence.
$F_{n}$	The current flight of the n-th heterogeneous graph in graph sequence
$D_{nm}$	The flight $F_{n}$’s neighbors with the same departure airport, m∈{1,2,3}
$A_{nm}$	The flight $F_{n}$’s neighbors with the same arrival airport, m∈{1,2,3}
$h_{i}$	Flight *i*’s features, $i\in \{F_{n},D_{nm},A_{nm}\}$
$h_{F_{n}^{d}}$	Aggregate feature of flight $F_{n}$ in the departure network
$h_{F_{n}^{a}}$	Aggregate feature of flight $F_{n}$ in the arrival network
$h_{F_{n}^{G}}$	Aggregate feature of flight $F_{n}$ in the whole heterogeneous graph
$g_{D}^{k}$	The soft gate to control the importance of *k*-th head in departure network
$g_{A}^{k}$	The soft gate to control the importance of *k*-th head in arrival network
$h_{s_{n-1,n}}$	The feature of sequence $F_{n-1}$ and $F_{n}$
$h_{G_{n}^{\prime }}$	Final embedding of Flight $F_{n}$

**Table 2:** The classification results of SGDAN with Equation (18).

Delay Time (min)	Precision	Recall	$F_{1}$
≤30	0.92	0.95	0.93
31–60	0.83	0.75	0.79
61–90	0.85	0.72	0.76
91–120	0.83	0.63	0.72
121–150	0.90	0.40	0.55
>150	0.91	0.45	0.60
total accuracy		0.89	

**Table 3:** The classification results of SGDAN with Equation (19).

Delay Time (min)	Precision	Recall	$F_{1}$
≤30	0.97	0.79	0.87
31–60	0.80	0.81	0.80
61–90	0.54	0.70	0.61
91–120	0.35	0.85	0.50
121–150	0.16	0.92	0.27
>150	0.20	0.95	0.33
total accuracy		0.79	

**Table 4:** The classification results of binary categories.

Model	Accuracy
[41] LSTM	0.88
[42] LSTM	0.87
[5] NN	0.73
[5] SVM	0.73
[5] RF	0.76
[16] GBDT	0.88
[15]	0.90
**SGDAN**	**0.91**

**Table 5:** The classification results of three categories.

Delay Time	[16] GBDT	[15]	SGDAN
≤30	0.778	0.806	0.925
31–120	0.855	0.889	0.841
>120	0.691	0.673	0.912
Total accuracy	0.795	0.814	**0.901**

**Table 6:** The classification results of four categories.

Delay Time	[16] GBDT	[15]	SGDAN
≤30	0.786	0.853	0.918
31–60	0.582	0.545	0.831
61–120	0.603	0.692	0.843
>120	0.723	0.719	0.904
Total accuracy	0.674	0.700	**0.894**
